# International Homœopathic Congress

**Published:** 1891-08

**Authors:** 


SESSION OF SATURDAY, JUNE 20TH.

### INTERNATIONAL HOMŒOPATHIC CONGRESS.

(From the *Public Ledger*, June 17th to 22d.)

The fourth quinquennial meeting of the International Homœopathic Congress was called to order on the evening of Tuesday, June 16th, by Dr. Richard Hughes, of Brighton, England, the permanent Secretary. Mayor Hoffman delivered an address of welcome, after which Dr. T. Y. Kinne, President of the American Institute of Homœopathy, made the address of greeting, and, as he referred to the name Hahnemann, a painting of the founder of this school of medicine was unveiled, after which he moved the organization of the Convention by the election of the officers, as heretofore printed in the *Ledger*, with the exception of Dr. Clarence W. Butler, of Montclair, New

Jersey, President of the International Hahnemannian Association, as one of the Vice-Presidents. Rules of order and an order of business were adopted.

Dr. Tisdale Talbot, of Boston, then took the chair and read the address of the Honorary President, Dr. R. E. Dudgeon, of London, England, whose age prevented his presence. A Committee on Business, with Dr. J. H. McClelland, of Pittsburgh, as Chairman, was appointed, and one on resolutions, with Dr. J. P. Dake, of Nashville, as Chairman. The Convention adjourned, to meet

WEDNESDAY MORNING, JUNE 17TH.

Promptly at ten o'clock, Dr. Talbot, of Boston, took the chair and announced that the address, as noted in the programme, would be delivered, the subject being

"THE ETHICAL BASIS OF THE SEPARATE EXISTENCE OF THE HOMŒOPATHIC SCHOOL."

Dr. Asa S. Couch, of Fredonia, New York, began his address, which was the principal and most important one of the day, upon the subject as stated above. He said that, to treat this subject satisfactorily, two things are primarily requisite: first, to define ethics and how its rules may be justly administered, and a comparison of our own with the drug therapeutics of the dominant school in medicine. Ethics, according to an eminent lexicographer, is defined as the "science of human duty," and who would administer thereupon must be ethical. A school, which should formulate decisions within the science of human duty, while uncertain of its own position or when inspired with passions and prejudices, would place itself in an unfortunate position before the world, and one likely to end in embarrassment. After a few more introductory remarks, he went on to say that, whether success or failure, life or death, follows the experimental administration of drugs, no logical inference can ensue, for they must follow each other in sequence and lap each other as results. To increase peristalsis where deficient, or to arrest it by drug poisoning where in excess; to force or diminish secretions; to accelerate or retard the circulation; to stop all voluntary and many involuntary activities and demand that it be called sensible or scientific doctoring, is a travesty upon logic and a caricature of common sense. In a large majority of instances such practice aborts the very process by which Nature would cure; in all cases it handicaps her by adding to her burdens and diminishing her power of resistance. Its futility is recognized by sufficiently intelligent and honest authors of the old school. In fact, its own writers have been its most severe and unsparing critics and their denunciations stand unchallenged before the world.

Referring to old-school treatment of certain diseases, he presented the matter to the audience, arguing against it, and stated that the embarrassment of the situation to such of the old-school brethren who can be embarrassed comes from the fact that they have no law by which to proceed in the prescription of remedies, and hence no more actual science than the Indian medicine man, who assays to cure by blowing feathers and beating tom toms. Whatever improvement may have obtained in old-school practice within two decades has been purely and altogether negative. Through the evolution of mind and the embarrassment of marked contrasts, it has increased its conservatism, and as the result of a kind of intellectual osmosis, imbibed from the doctrine, process, and results of a school founded by an inhibited Saxon, it has lessened its doses and diminished its polypharmacy, but in its principle or doctrine of medication it remains absolutely unchanged. Even the purloinings from Homœopathy, as embodied in the works of Ringer, Phillips, and others, have not greatly modified its practice.

First. Because a large majority of its practitioners have no recourse to these works.

Second. Because in so far as they have been successfully adapted, it is not their legitimate practice, it is that of a slipshod and very crude Homœopathy.

Without fear of successful contradiction the speaker went on to say the principle of honest allopathic practice to-day is not one whit in advance of that of pre-historic man, nor in anyway changed except by the unfortunate doctrine of the illustrious

Galen. It is without any law whatever, and consequently the application of the term science in relation to it is a misnomer and a dishonor to the word. Yet consider the amount of drugs that is being poured into mankind and reflect upon the indorsement it receives.

During the last customs year at New York, there were imported for medicinal use of the aqueous extract, tincture, and other liquid preparations of opium, twenty-nine pounds; of morphia and all salts thereof, sixteen thousand six hundred and twenty-nine ounces, and of crude opium, containing nine per centum and over of morphia, two hundred and thirty-three thousand six hundred and fifty-five pounds. This is one port. When the importations at the others are figured up, what must be the aggregate cast upon our shores? Time will not permit a sufficient analysis of the matter, but I may ask you who know what its curative application is, and in what doses it is effective, to consider, except in proper palliation, or by those who have acquired a horrible habit through its abuse as medicine, how the rest of the vast amount has been, or will be, employed.

Man is but a system of reflexes. Either in health or disease to embargo the one is to arrest the other, and this, except under law to cure, is what by scientific (?) application this opium (or its salts) has been or will be doing throughout the land, masking disease, lessening healthful resistance, and deceiving unfortunates who have trusted themselves to the tender mercies of an arrogant and self-sufficient school.

Last spring, in a given time, the registry of vital statistics in the city of Buffalo recorded the certificates of death from pneumonia, bronchitis, and la grippe as numbering seventy. Of these, sixty-three were from allopathic and two from homœopathic physicians. Of the old school there are three hundred and of the new school sixty physicians in that city. Multiplying the number of deaths under homœopathic treatment by five to keep the proportion just, and the result is as ten to sixty-three, and subsequent investigation proves a constrast still more startling.

Referring to Hahnemann, the speaker said he had character enough to scrutinize and analyze that which he was commanded to do with poisonous drugs, and sense sufficient to hesitate before doing it.

He said, however, he was not weak enough to stand and declare that this school embodies an absolute science in therapeutics. No school which ever may or can be founded will do this.

The fully-prepared practitioner in this school does not guess; he does not experiment; he does not deliberately set to work to make his patient sicker. The law under which he shall proceed is one in nature and results obtained in exact application. When an epidemic appears he does not grope in the dark and try experiments unto the death of thousands. Given the symptoms in advance, he can even foretell the remedies which will successfully grapple with a coming scourge.

After contrasting the two schools in medicine, the therapeutic methods of one devoid of danger to the sick and adapted to comparative certainty in the application, those of the other without law, and consequently fraught with menace to mankind, he continued: On the showing, can there remain doubt as to the ethical basis of the separate existence of the homœopathic school?

In concluding, he referred to the discrimination against homœopathic physicians by insurance companies, also by the Government, and the exclusion of their school from the army and navy. Referring to the old school's hold upon the Government, he said that school is striving to enter the Cabinet. It wants a "Secretary of Health," and it will exert all its arts and all its power to secure one.

He said the homœopathic profession appears to be insensible to the fact that every assumed superiority unprotested and every important position appropriated by the old school holds prestige for that school, and casts the shadow of unequal value upon its own.

Reference was made to the strong intrenchment of the old school, and an anecdote of General Grant was recited. During his administration the Commissioner of Pensions began discharging examiners who had the courage to affirm their belief and practice of Homœopathy. The matter brought to the President's attention, the next head going into the basket was that of the Commissioner M. D.

Concluding he said, duty, duty, duty should be inscribed all over the simple creed, "*Similia similibus curantur*;" duty to organize in readiness for resistance or proper aggression; duty to subordinate modesty and profit when public positions may be secured conserving the interests of the school; duty to educate the public mind against empirical practice and the masking of disease through the physiological power of drugs, and duty to patronize the journals of the school, that their influence may be extended.

To do this most effectually we must as individuals purge from our lives all taint of personality, affectation or superiority, and uncharitableness. We must be thoroughly imbued with the high importance of our mission, and pursue it only in the spirit of justice and benevolence. Standing before the world in this wise—exalted in the forceful and honorable character thus derived, we shall occupy a position like that commanded by Him whose goodness and mercy are the blessing of the world. "Let your light so shine before men that others seeing your good works may follow them."

### INFLUENCE OF HOMŒOPATHY.

Dr. A. C. Cowperthwaite, of Iowa City, Ia., then read a paper, "The Result and Influence of Homœopathy upon the Theories and Practice of the Medical Profession."

Dr. Richard Hughes, of Brighton, England, then read a synopsis of a paper prepared on the same subject by Dr. S. Lilienthal, of San Francisco, Cal., and discussed it briefly.

The paper, "How to Cure Backache," prepared by Dr. Edward T. Blake, of London, England, was read by Dr. T. Y. Kinne. In introducing the subject he asked, "Is the backache due to local functional change; is it the result of local organic disease; is it a topical expression of general diathesis, or is it merely a reflex from a distinct disorder in another part?" The diagnosis must be deliberate, and the elements of accuracy in making the diagnosis were given, and also the tests of disease. In discoveriug the disease the more probable causes of backache must not be forgotten; a bent whalebone or the button on a heavy skirt in a woman or in a man, a non-woolen trouser waistband soaked with sweat, and causing resultant chill. After practicing usual crural and abdominal reflexes, direct the patient to arch the back and rest on occiput and heels; request the subject to walk in a straight line, eyes shut, and at the same time to play an imaginary fiddle. An unexpected faradic shock applied to the loins will cause involuntary opisthotonos in a malingerer or in a "malade imaginaire." Some special curves disappear on patient "dressing up" vertically and trying to look square.

In the discussion that followed Professor Snyder, of Cleveland, said that every one attempting to cure backache with electricity should understand it. Often harm is done by the ignorant use of the remedy. Where symptoms correspond very closely better results are obtained by the homœopathic system.

Dr. Monroe, of Louisville, Kentucky, referred to an amusing case. The patient, having suffered from spinal irritation and nervous prostration, grew worse from time to time, till digestion was affected, numbness in hands and feet, hysterics, sleeplessness, resulted; tried all physic; went to general practitioner, but kept declining. Finally the patient, who was a woman, broke her corset string and had to get a new one. In two weeks she was well, as the knot on the string pressed on exit of spinal nerves.

Mrs. Harriet J. Sartain, M. D., of Philadelphia, suggested that many a backache was due to mechanical causes. If there were no corset strings there would not be so many backaches.

Dr. Brewster, a woman practitioner, of Baltimore, said that backache was due to deficient circulation. Her treatment, while not a profitable one, perhaps, for the physician, was merely a hygienic one. She first taught her patient how to breathe. Backache was caused by too little exercise. You must not depend on drugs, but the cure must be brought about by amending the habits of life.

Dr. Pemberton Dudley referred to a patient coming to him for intercostal neuralgia. He was troubled on Sundays only, and it was found on that day of the week he carried a heavy silver watch, pressing on nerves affected.

The subject, "Homœopathy, in its relationship to Constitutional Predispositions to Diseases," was treated by Dr. Aug. Korndoerfer, of Philadelphia.

Dr. James H. McClelland, of Pittsburgh, followed with a paper, prepared by Dr. P. Diederich, of Kansas City, who was too ill to deliver it, on the subject of "Homœopathic Medicines as Prophylactics and Homœopathic Constitutional Treatment." Only a synopsis was read, and a discussion ensued, participated in by Drs. Allen, of New York, and Morgan, of Philadelphia.

"The Import of Bacteriology in Homœopathic Therapy in General" was the subject assigned to Dr. Walter Y. Cowl, of New York.

The reading of this paper closed the morning session, and discussion was put over until the afternoon, but before adjourning President Talbot read telegrams of congratulation and greeting from the President of the Homœopathic Medical Association, of Germany, and from Dr. Lilienthal, of San Francisco, a distinguished author and physician. The Treasurer, Dr. Kellogg, of New York, who is recuperating broken health in Mexico, also sent words of greeting.

AFTERNOON SESSION.

Dr. Walter Y. Cowl, of New York City, continued his paper on the "Import of Bacteriology to Homœopathic Therapy." Dr. Alexander Villers, a prominent homœopathist of Dresden, Saxony, and editor of the *Homœopatische Zeitung*, expressed his views and advocated a thorough examination of the patient by the physician, instead of taking, as generally is the case, the patient's own version of the symptoms.

Dr. J. Nicholas Mitchell, of Philadelphia, took as a subject, "Is Antisepsis Called for in Obstetrical Cases?" and delivered it in an able manner. The discussion was opened by Dr. C. G. Higbee, of St. Paul, Minn., with a short talk on the bacteria theory in puerperal fever, and advised homœopathists to use aseptic measures. Dr. Bushrod W. James said that it must be remembered that diseases come in many ways, and that in the treatment of cases the entire case must be individualized by the homœopathist, whether puerperal fever or other, and the entire symptoms of the case must constitute the basis on which the prescription is given.

Dr. I. Tisdale Talbot, the Chairman, referred to a cablegram received from the French Homœopathic Society, extending greeting to the Homœopathic Congress. It was unanimously voted that the Secretary be instructed to send a message, thanking the French homœopathists for their interest, to Mr. James Love, Secretary of the Society.

The paper on "Pregnancy," by Dr. Emily V. Pardee, of South Norwalk, Conn., was responded to by Dr. Millie J. Chapman, of Pittsburgh, who particularly emphasized the importance of dress and diet.

### SESSION OF THURSDAY, JUNE 18TH.

At the appointed hour Dr. Talbot took the chair. The programme was headed "Materia Medica Day," and the interest in the subject was indicated by the large attendance present. The principal address was made by Dr. Jabez P. Dake, A. M., of Nashville, Tennessee, and the subject was: "Civil Government and the Healers of the Sick."

### DRUG PATHOGENESY.

The report on the Cyclopædia of Drug Pathogenesy, by the editors, Dr. Richard Hughes, of Brighton, England, and Dr. J. P. Dake, of Nashville, Tennessee, was read by Dr. Hughes. He stated that the work was practically finished. The principal circumstance leading up to the beginning of the work was that the materia medica of Homœopathy had long been scattered in divers languages. In 1876 Dr. T. F. Allen, of New York, undertook to remedy the defect, and in six years presented our whole pathogenetic wealth. The possession thereof only accentuated dissatisfaction, and the editor himself revealed so many flaws in the execution that the conviction forced itself on most minds that the work should be done over again on a more critical and better plan.

Dr. Hughes continued that America was looked to for a translation of Hahnemann's *Chronic Diseases* worthy to rank with that of the *Materia Medica Pura* of Great Britain.

Dr. Woodward, of Chicago, congratulated the editors on the completion of the work. Professor T. F. Allen added his testimony to the value of the work, and Professor C. S. Mack, of Ann Arbor University, dwelt upon its reliability, while Dr. Pemberton Dudley also added words of praise for the effort.

### DEMANDS OF MODERN SCIENCE.

A résumé of the "Demands of Modern Science in the Work of Drug Proving," by C. Wesselhœlft, M. D., of Boston, was read by Dr. A. C. Cowperthwaite, of Iowa City.

Dr. Hughes, of England, then followed on the subject, "Drug Proving of the Future." What shall be proved, and how shall the proof be made? were the two questions the speaker stated it would be his desire to answer. The selection of drugs for the future should be guided by their usefulness as remedies. He upheld the facts accepted by the school, and maintained that Hahnemann's dynamization, however baseless the theories about it, is a fact. Attenuation, when conducted according to his method, does more than simply weaken virulence. In some cases it develops energy; such energy cannot be limited to the therapeutic sphere, but may, at any rate on some subjects, display itself pathogenetically also, and in actions unknown to the crude drug. The speaker recognized that special care must be taken to avoid illusive, and for the elimination of the working, expectant attenuation. Potencies will produce medicinal effects which crude drugs cannot excite, which we, as heirs of this discovery of Hahnemann, must not neglect. The symptoms thus obtained are of a class especially suitable to homœopathic practice. He finally urged that, as homœopathists, the proving of drugs on animals should not be entirely left to the old school, as their procedure rarely subserves our ends.

Dr. T. F. Allen, of New York City, spoke in the discussion following this paper, and was strongly inclined to the establishment of laboratories, where the improvement and proving of the drugs might be made a specialty. He referred to the offer of a wealthy man who had promised to make such an institution possible.

Dr. Charles Mohr, Professor of Materia Medica in the Hahnemann College, paid a tribute to the excellence of the papers read, making the declaration that unsystematic proving of drugs was to be discountenanced. Laboratories, he contended, would greatly help in attaining the results desired, in which view Dr. Dake coincided. He said that hospitals and colleges were good, but the root of all was the proving of drugs.

Dr. Sutherland, of Boston, editor of the *New England Medical Gazette*, joined in the discussion, as did also Dr. Morgan, of Philadelphia, and Dr. Van Denberg, of Fort Edward, New York. Adjournment was here made for dinner.

### AFTERNOON SESSION.

Dr. J. Wilkinson Clapp, of Brookline, Mass., opened the afternoon session with a paper on "The Pharmacy of Triturations." The best methods were explained by which trituration could be proceeded with without destroying the properties of the drugs, or adulterating them with the alkali of the material of which the mortar or pestle is composed. Trituration with coarse material and several hours' action will not bear as good results as a finer preparation, for a shorter time, with the proper quantity of sugar.

An essay entitled "The Pharmacy of Tincture" was rendered by Dr. Lewis Sherman, of Milwaukee, Wis. He spoke of preparation of homœopathic tinctures. The tincture must tell the truth, and nothing but the truth. It must be free from any taint or trace of the property of any other substance than its competent parts.

### THE PHARMACY OF TINCTURES.

Dr. E. M. Howard, a physician, of Camden, read the substance of the paper prepared by Mr. A. J. Tafel, of Philadelphia, entitled "The Pharmacy of Tinctures." In discussing the matter the doctor claimed that the English method was more accurate, but only a relative quantity of accuracy can be obtained, as plants at different seasons contain different quantities of tinctures. Hahnemann, the doctor said, was right when he adopted fresh plants as tincture bases. Accuracy was more nearly obtained by tracing tinctures on dried plants. What is wanted in tincture is not so much the quantity, but the strongest possible solution in the most powerful form. In the future the aim in tincture making should be not uniformity, but to obtain strongest solution from any given plant.

Dr. T. F. Allen, of New York, then followed in an address, "Indexes and Repertories," and said it was of the highest importance that a feasible method of indexes should be adopted. It was to the homœopathist full of valuable information and suggestion, and showed care and ability in the preparation.

Dr. Mohr, of Philadelphia, opened the discussion which followed. He strongly favored indexing all symptoms in contradistinction to the desire of some to eliminate the minor symptoms. He also suggested that the symptoms be arranged in the concordance style of indexing.

Dr. Charles S. Mack, of Ann Arbor, Michigan, read a paper on "Discussion of Dr. Hughes' Proposed Index to the Cyclopædia of Drug Pathogenesy." He advised the use of three figures in the indexing, the first to indicate the number of provers of the symptoms; the second, number of times symptoms appeared, and third, in how many cases of poisoning was it prominent.

Dr. M. W. Van Denburg, of Fort Edward, New York, opened the discussion, and explained that the method requiring the least time and the least expenditure was the most desirable. Dr. Charles A. Church, of Passaic, N. J., said that his method was to index on the margin of his Materia Medica, and under what drug the remedy was to be found.

Dr. Augustus Korndoerfer, of Philadelphia, did not believe that a repertory will ever be obtained that will suit every one.

Others who discussed the subject were Dr. John C. Morgan, of Philadelphia; Dr. J. B. Dake, of Nashville, and Dr. Richard Hughes, of Brighton, England.

An instructive essay on "A Reconstructed Materia Medica," by Dr. Price, representing the Baltimore Medical Investigation Club, was very interesting, and was discussed at some length by Dr. Dake and Dr. Sutherland, of Boston, editor of the *New England Medical Gazette.*

An extract of an essay relating to "The Probable Homœopathic Uses of Some New but Unproved Drugs," by Dr. E. M. Hale, of Chicago, was delivered by Dr. Richard Hughes.

Dr. M. W. Van Denburg, of Fort Edward, N. Y., had prepared a paper on "A Comparison of Therapeutic Methods Based on a Study of Arsenic," but, at his own request, was excused, and the document passed to the Committee without reading.

The subject of "Pharmacy," which had been left unfinished at the morning session, was again taken up, and was discussed by Dr. T. C. Duncan, Dr. Richard Hughes, Dr. James H. McClelland, Dr. Lewis Sherman, and Dr. Pemberton Dudley. Some very interesting questions were developed during the debate, several of which were exceedingly instructive.

### SESSION OF FRIDAY, JUNE 19TH.

The President, Dr. I. T. Talbot, of Boston, opened with a paper on "The Duties and Responsibilities of Homœopathic Colleges as Leaders in Medical Progress."

A paper was read by Dr. James C. Wood, of Ann Arbor, Michigan, on the subject, "Epilepsy as a Hysteria Neurosis."

The discussion following was engaged in by Dr. Villers, of Dresden, Saxony, and Dr. Helmuth, of New York.

Dr. L. A. Phillips then followed, in the essay entitled "The Aids to Gynæcology, Medical or Surgical." In a résumé he suggested that gymnastic exercise was a superior remedy; also, obviating the pressure and weight of clothing, postural treatment, mechanical contrivances, which act as splints. Remedies recommended for internal use are very beneficial for external appliance.

Electricity was very potent for either good or evil, as a remedy, according to the care and discretion used in applying.

Dr. Danforth, of New York, spoke in regard to the development of electricity as a remedy, but it must be carefully handled.

Dr. Julia Holmes Smith, of Chicago, gave some pertinent professional suggestions from a woman's standpoint, she being followed by Dr. McClelland, of Pittsburgh, and Dr. J. C. Morgan, of Philadelphia; Prof. Snyder, of Cleveland, and Dr. Brewster, a female physician of Baltimore, the author of the paper, closing the discussion.

Dr. B. Frank Betts, Professor of Gynæcology in the Hahnemann College, read a paper on "The Scope of Homœopathic Therapeutics in Gynæcological Practice." He placed great stress on diagnosis of disease, suggested many new remedies, and advised preventive measures. He denounced astringent solutions, recommending cleanliness, exercise, and general hygienic measures; too much local treatment is bad. He considered surgical operations as necessary in many cases.

Dr. Johnson, a female physician, of Philadelphia, followed with a paper in discussion. Dr. Dake spoke to the subject, as did also Dr. Bushrod W. James, and the discussion was closed by Dr. Betts.

### AFTERNOON SESSION.

At the afternoon session the paper read by Dr. Ostrom was discussed.

Then followed the paper by Dr. J. M. Lee, of Rochester, N. Y., the subject being "Forty-seven Laparotomies in Two Weeks." As chief surgeon of the Rochester Hospital, the Doctor operated on this exceptionally large number of difficult cases, and it was received with great interest and was discussed in an animated way.

Dr. Chester G. Higbee, of St. Paul, Minn., next held the attention of the profession on the subject, "Gynæcological Surgery—when to Operate."

In the Department of Ophthalmology, Otology, and Laryngology, Dr. D. A. MacLachlan took the subject "Similia in Eye, Ear, Nose, and Throat Diseases." The Doctor is a Professor in the University of Michigan.

Dr. A. B. Norton led in the discussion which followed, and subsequent thereto.

### HAY FEVER.

Dr. Horace F. Ivins, of Philadelphia, read an essay entitled "Pollen Catarrh—Hay Fever."

Dr. Edward B. Hooper, of Hartford, Conn., delivered an interesting essay on "The Surgery of the Nose and Nasal Pharynx." The subject was discussed by Dr. N. A. Dunn, of Chicago; Dr. Bushrod W. James, and Dr. G. C. McDermott, of Cincinnati, O., Professor of the Eye and Ear Department in Pulte Medical College. A paper on "Points in Diagnosis of Muscular and Defective Eye Troubles" was read by Dr. Hayes C. French, of San Francisco, Professor Eye and Ear Diseases in San Francisco Homœopathic Medical College. "A Study of Ophthalmic Therapeutics" was discussed by Dr. F. Park Lewis, of Buffalo, N. Y., and the discussion was indulged in by Dr. McDermott, of Cincinnati, who spoke very energetically, and was frequently interrupted with applause; Dr. Korndoerfer, Dr. Wesley A. Dunn, and Dr. Bushrod W. James. Several papers prepared by Dr. Hayes C. French, of San Francisco, were, by his own request, referred to the Committee on Publication.

### SESSION OF SATURDAY, JUNE 20TH.

At ten o'clock the Institute adjourned, and the body resolved itself into the International Congress, Dr. Talbot in the chair.

Dr. Kinne, President of the Institute, offered resolutions, inviting the President of the United States to be present during its sessions, and at the banquet on Monday evening, June 22d.

"The Influence of Homœopathy on Recent Medical Literature and Practice" was the subject of the leading paper offered at the morning session, by Dr. Chas. Gatchell, of Ann Arbor, Mich.

The contention of the speaker was that there are few modern old-school text-books on materia medica and therapeutics that do not contain material gleaned from homœopathic works of a like character, and quoted Ringer, Phillips, Brunton, and Bartholow in testimony of his assertion.

Of the drugs that the old school has adopted from homœopathic sources, Aconite is the chief, and was one of the earliest they appropriated, and the one they most frequently use. Want of candor was the indictment the speaker found against the old-school writers for failing to give due credit to Homœopathy as being the source of their knowledge.

Homœopathy, the speaker continued, has a marked influence on the literature of the old school, but a consideration of the available evidence goes to show that a different verdict must be rendered in respect to its influence upon their practice. That Homœopathy has had the effect of compelling the school of traditional medicine to abandon to a great extent its harshest measures, and to reduce somewhat the size of the dose is true and well known ; but that it has had the desired effect of causing them to substitute Homœopathy for their former methods is a proposition that cannot be successfully maintained. Homœopathy has modified the old-school practice, but not in the direction of Homœopathy. Evidence was presented that homœopathic prescriptions were not made in old-school hospitals, or other institutions. The question was asked, if the old school is making any practical application of Homœopathy. No better opportunity ever presented itself than was offered by the recent scourges of epidemic influenza, the speaker continued. The disease fairly invited comparison of the similar remedy, and in the hands of homœopathic physicians was successfully treated with Gelseminum, Eupatorium, Arsenicum, Bryonia, Tartar emetic, and other well-selected remedies.

Not so the old school on the treatment of this disease. They brought to bear the most active measures taught by antipathy, empiricism, and physiological medicine. The Doctor stated that the members of the old school of medicine are not making use of homœopathic methods in the treatment of the sick.

Concluding, the speaker said that the great school of traditional medicine is in close contact with Homœopathy; it is placed within their easy reach, and yet members of that school fail to make practical application of homœopathic therapeutic methods, and the failure lies in the fact that old-school physicians attempt to practice Homœopathy empirically. That is impossible to do; there is no royal road to our therapeutical methods. The empiricist tries to find one and fails, and abandons further effort. If Homœopathy were capable of empirical application in practice, the old school would have taken complete possession of it years ago.

The final statement of the speaker was: In practice our methods are as safe from their unacknowledged appropriation as if our rights were guarded by statute law, for the reason that they have not learned the true secret of the successful homœopathic prescription—differentiation of the remedy and the individualization of the case. This is done by no one recognized as an old-school physician, nor will it ever be, for whenever one of their number goes so far he ceases to be an old-school physician. From that time he is a homœopathist. Soon this man makes a confession of faith, he shows his belief, and swears allegiance to Hahnemann. Each year their number equals the combined number of graduates from all colleges. In this way are our ranks recruited.

The paper was received with evident satisfaction, and Dr. Holcombe said that all were delighted when homœopaths see approximation of the old school to Homœopathy.

### ANTISEPTIC METHODS.

The morning programme was devoted to essays and discussions relating to surgery, and Dr. Horace Packard, of Boston, led off with the subject, "The Present Relations of Antiseptic Methods to Surgery." Dr. Lungren, of Toledo, Ohio, a prominent specialist, in discussing it, said the antiseptic treatment as practiced at the present day is entirely unnecessary. Dr. Sheldon Leavitt, of Chicago, stated that at Berlin he had witnessed operations conducted under the antiseptic method used by Tait.

"Carcinoma and Sarcoma" was the subject of a well-received address by Dr. Wm. Tod Helmuth, of New York City. He strongly denounced technical papers. His argument was the direct result arising from a careful study of one hundred consecutive cases. He explained the difference in formation of the two malignant diseases, cancer and sarcoma, explaining that they may sometimes exist simultaneously in the patient. The paper was comprehensive and original, and reviewed in detail various remedies which, in the experience of the speaker, had proved useful, prominent among them being the so-called Arsenic cure. Other forms of the disease are frequently cured by Hydrastis.

A lengthy discussion, in which all the points were canvassed and commended, ensued, in which two facts in particular were strongly brought out: That intestinal surgery had its American origin in the personal work of Dr. G. D. Beebee, of Chicago, a quarter of a century ago. In proof of this claim Dr. Helmuth cited a case in which five feet of the smaller intestines were removed with complete success. The other point being that the much lauded Phenic acid, which emanated from Paris, a few years since, was introduced by the same physician, and at the same time as described in the foregoing case.

Dr. W. B. Van Lennep, a professor in the Hahnemann Medical College, of Philadelphia, and one of the editors of the Hahnemannian *Monthly*, addressed the Congress upon the subject, "Inflammation of the Right Iliac Fossa." This subject he pleasantly condensed under the head Appendicitis, after which he explained the causes, course of development, pathology, and complications of this dread malady.

The importance of the paper centered upon the question of when and how to operate in such cases, and how much the rate of recovery was thereby increased. These questions were all answered in a clear and forcible manner.

The paper was discussed by Dr. J. E. James, of Philadelphia, who took one exception to the paper, and that was that the restriction to the one condition mentioned would to many minds be misleading. He advanced the avoidance of undue surgical interference on one hand, and extreme conservatism on the other. He believed that consistent homœopathic treatment is capable of curing many severe cases that are now being operated upon.

A general discussion followed until the one o'clock adjournment.

Just before adjournment a resolution was passed requesting Dr. R. E. Dudgeon, of London, England, the honorary President of the Congress, to prepare a new edition of Hahnemann's *Organon*, also to secure translations of such as yet unpublished papers of value as were in his possession.

AFTERNOON SESSION.

A paper, the subject being "Training School for Nurses," prepared by Dr. Henry Minton Lewis, of Brooklyn, was read by Dr. Kinne in the absence of the author. The specifications for a good nurse, it was set forth, should be, first, good health; second, comeliness, must be practical, not emotional, discreet, and observing and honest; third, nurses must have good education, must write plainly, so that records may be read easily; must read well, and be able to talk intelligently.

In England, it was claimed, the training schools educate two classes of nurses. One class includes those who propose making the profession their means of livelihood; another class those to have charge of hospital and mission work.

The discussion was opened by Dr. Julia Holmes Smith, and she stated that the requirements of a nurse are that she must be a perfect woman. She must keep on good terms with the doctors and be patient with the patients. There should be a good normal school somewhere to teach all nurses.

Dr. John L. Moffett, of Brooklyn, editor of the *North American Journal of Homœopathy*, advocated nurses of a high standard.

Dr. T. C. Cook, of Buffalo, quoted some personal observations, and said that nurses are more attentive to their work and often superior when graduated from homœopathic hospitals.

Dr. D. H. Beckwith, of Cleveland, believes that a good nurse is more important than a doctor at the bedside.

Dr. William Owens, of Cincinnati, read a paper on "The Relation of Hygiene, Diet, and Therapeutics to Morbid Conditions of the Alimentary Canal of Infants."

There was a discussion by Dr. W. F. Edmundson, of Pittsburgh, and Dr. Custis, of Washington.

Dr. D. G. Wilcox, of Buffalo, followed in a paper, "Surgery of the Spinal Cord," then came Dr. E. A. Pratt, of Chicago, on "Orificial Surgery," in which discussion was engaged in by Dr. A. L. Monroe, of Louisville, Ky.; Dr. Wm. Tod Helmuth, of New York; Dr. Eugene F. Storke, of Colorado; and Dr. H. P. Skiles, of Chicago.

Dr. John C. Morgan favored the use of the liquor from corned beef and cabbage for cases of cholera infantum, and it could be used on infants as young as ten days.

The Convention then adjourned to meet on Monday morning.

### SESSION OF MONDAY, JUNE 22D.

The report of the Intercollegiate Committee of 1891, by Dr. I. Tisdale Talbot, its chairman, was presented.

The growth of Homœopathy in the past five years was the subject of the address of Dr. T. Franklin Smith, of New York.

### MEDICAL LEGISLATION.

Dr. Dake presented the report of the Special Committee on Medical Legislation.

Reports on the condition and progress of Homœopathy in various countries were next in order, and brief summaries of the reports follow:

Dr. Richard Hughes, of Brighton, read the English report, which was prepared by Ernest H. Stancourt, M. B., C. M., Southampton, England. It only spoke in a general way of the progress made, but it was claimed this was very gratifying.

The paper on Australia was likewise presented by Dr. Hughes, and there it was shown that Homœopathy was steadily progressing. The death-rate on typhoid fever cases was quoted. In old-school hospitals the death-rate was thirteen per cent., and in homœopathic hospitals eight per cent. In Tasmania there is great encouragement for the speedy establishment of a hospital being accomplished.

The New Zealand report was prepared by John Murray Moore, M. D., F. R. G. S., but read by Dr. Hughes, and it stated that in all the free, self-governing colonies of the British Empire the homœopathic system, when represented by qualified men of ability and respectable character, has established itself firmly in the confidence of the people—a people more quick and intelligent than in the parent country.

From India a report was written by P. C. Majaindar, L. D. S. The history of Homœopathy in India since the last International Congress was full of events for continued progress and improvement, and the Hahnemann method has gained an entrance into all the nooks and corners of this country.

Of Denmark, Oscar Hansen wrote that there Homœopathy was not known until 1831, when Hans Christian Lund introduced the system. There the Homœopathic Society has now one hundred members.

From Mexico, some news was sent by Dr. Joaquin Gonzales and read by Dr. Kinne. Homœopathy was there introduced in 1850. A six-year course is there required instead of four in this country, and this particular school of medicine has equal rights before the law, and cholera in that country has been successfully combatted by homœopathic treatment.

In Switzerland, according to Dr. Th. Bruckner, the report was of rather a negative character. The poor take advantage of the sick funds, and are attended by physicians employed by the officers of the funds, and the advantage is against the homœopath.

Dr. A. Von Villers, of Dresden, read the report from Germany. Losses by death of eminent physicians were reported; also the opening of a new hospital at Leipsig, capable of accommodating two hundred patients, as well as the addition of many new physicians to the ranks of Homœopathy. The report stated the requirement of the Government, at Wurtemberg, to the effect that every student of medicine shall have a sufficient knowledge of Homœopathy to be examined in it.

In Austro-Hungary no change has been noticed since 1886. The right to dispense their own medicines has again been accorded homœopathic physicians by law of May 27th, 1887.

In Austria Dr. Fr. Klauber writes that the homœopathists are completely thrown on their own resources, as all the legacies and bequests aiming at the establishment of a homœopathic chair in Vienna University have been disregarded.

For five years the school has remained stationary. The patients of Homœopathy are scattered, but mostly the nobility, by birth as well as by education, owe allegiance to this method of healing, to the discomfiture of our powerful opponents enjoying the guaranty of the State.

Dr. A. Lorbacher, of Leipsig, wrote that, in Germany, continual aggression was maintained, the whole of the land being dotted over with a network of homœopathic societies, and the Hahnemann principles are in no danger of going under in their mother country. From all offices or employments in the army and hospitals homœopathists are excluded, and are not allowed to explain themselves in the homœopathic press.

In Wurtemburg the large Society of Hahnemannia enjoys the protection of Queen Olga, and counts among its members persons of high standing and of the best families. There are, altogether, six hundred doctors, and about fifty have, in the past five years, passed the Prussian examination for dispensing.

From Russia the report was read by Dr. Richard Hughes as prepared by Dr. Bojannus, of Moscow. It is stated that in St. Petersburg a fund of over one hundred thousand roubles has been raised to build a hospital, and land for the same has already been granted by Imperial order.

The following answer to the invitation tendered President Harrison was received:

### PRESIDENT HARRISON'S COMPLIMENTS.

Cape May Point, June 21st, 1891.

Pemberton Dudley, M. D., Secretary, etc.

Dear Sir:—I beg to acknowledge by the hands of my friend, Dr. Gardner, the invitation of the International Homœopathic

Congress, now in session at Atlantic City, to visit the Convention and to attend the banquet to be given to-morrow evening. Will you be good enough to express to the Convention my high appreciation of its kindness and my regret that arrangements already made render it impossible for me to accept the invitation? With great respect, very truly yours,

BENJ. HARRISON.

Dr. Bushrod W. James spoke on the subject of the progress of Homœopathy, as ascertained from the reports of the different countries, and especially in the United States.

Dr. Pemberton Dudley offered resolutions protesting against professional ostracism by the old school. They were referred to the Committee on Resolutions.

HOSPITALS.

An address was delivered by A. R. Wright, M. D., of Buffalo, N. Y., on "Hospitals—their Construction, Maintenance, Management, etc." The speaker contended that air and light were important considerations to be thought of in the selection of a site. As for the healthy these are important requisites, so also are they doubly so to the hospital patients. A spacious lot is necessary above all things for the prosecution of successful hospital work. Of the buildings, temporary or permanent, he said: "If the walls are thoroughly and solidly built, and finished with no reasonable chance for cracking and no air space in them, and so perfectly finished that no foul effluvia may find permanent lodgment in them, destruction and rebuilding seem unnecessary. A proof of this is found in the old Pennsylvania Hospital, bearing the date on its front of 1755. These walls were so well constructed that they are now considered satisfactory, though one hundred and thirty-five years old. The army hospital must necessarily be temporary, but the city hospital, with all present available means for perfect construction, should be so well furnished in walls and interior work that it may, in effect, be a permanent structure." Brick is preferable to stone for walls, as is also the general plan of isolated pavilions. The block system was worthy of consideration, but it does not, from its arrangement, allow as free circulation of air, and unobstructed light as in the pavilion system, therefore the consensus of opinion of the best men, authorities on hospital construction, favor the pavilion plan.

The speaker went on in matters of detail regarding hospital furnishings and the like, and, in conclusion, suggested that in all cities of twenty thousand people homœopathic physicians organize a hospital association, and begin work at once for a building fund for a hospital. " A well-equipped institution, be it college or hospital, will add greatly to the prestige of the profession in the community. If there should be an allopathic hospital, the strife should be to keep abreast of it. If there be none, let Homœopathy take the initiative."

The paper was followed by a general discussion.

At the afternoon session the Committee on Resolutions reported that they had considered the resolution offered by Dr. Pemberton Dudley and recommended its adoption as the sentiments of the Congress. Dr. Richard Hughes, as Chairman of the Committee on deciding the place of next meeting, announced that it would be in England in 1896.

A learned paper on the "Treatment of Insanity" was read by Dr. N. Emmons Paine, of Westborough, Mass. The speaker advocated the "rest treatment" as a cure for insanity, the six elements of which are seclusion, rest, diet, massage, electricity, and therapeutics. The diseases to which the speaker said they can be applied with a hope of cure are locomotor ataxia, uterine disease, chorea, hysteria, neurasthenia, insanity.

Dr. S. H. Talcott, of Middletown, N. Y., delivered an essay on the same subject, entitled, "The Curability of Insanity by Homœopathic Medication."

These papers were indorsed by Dr. H. B. Fellows, of Chicago; Dr. J. C. Morgan, of Philadelphia, and Dr. Wanstall, of Baltimore.

Dr. Salger, of Calcutta, India, sent a paper, entitled "Asiatic Cholera," and treating of this dreaded disease. It was read by Dr. Richard Hughes. Dr. E. M. Howard, of Camden, read Dr.

H. M. Dearborn's essay on "Lanolin and Aquine in Diseases of the Skin." Dr. Martin Deschere, of New York, being absent, a paper, entitled "Diet and Homœopathic Treatment," was read by Dr. J. H. Gann, of Worcester, and two papers by Dr. Gailliard, of Brussels, Belgium, "Hahnemannian Remedies of Chronic Diseases." Dr. Eugene F. Storke addressed the Convention on the "Climatic Cure of Colorado," he being followed by H. R. Stout, of Jacksonville, Florida, on the "Climate of Florida."

Dr. J. P. Dake, of Tennessee, offered resolutions of thanks to the publishers of the various newspapers publishing the business of the Congress and to the correspondents for their work. The Committee on Resolutions also presented the following, which was unanimously adopted: "*Resolved,* That our thanks are due and are hereby tendered to Mr. George W. Childs, publisher of the *Public Ledger*, for the complimentary copies of that most valuable newspaper." The thanks of the Convention were tendered the various officers and committees for their ability and careful work, and the entire assemblage sang the Old Hundred Doxology, and the Congress stood adjourned, the next meeting to be held in England in 1896.

The American Institute of Homœopathy then met, passed resolutions of thanks to their officers, and soon after six o'clock their session closed, to meet again in Washington, D. C., next June.

